# Chronic Aphonia Cured during an Attack of Pneumonia

**Published:** 1864-04

**Authors:** Thomas Kinchley

**Affiliations:** Assistant Surgeon P. A. C. S.


### ART. V.—*Chronic Aphonia cured during an attack of Pneumonia.* By THOMAS KINCHLEY, Assistant Surgeon P. A. C. S.

I respectfully offer the following report of a case pneumonia, connected with an aphonic affection of some ten months' standing. I do not offer the case as one possessing any extraordinary merit, but because of the happy result to the patient in recovering his speech under such singular circumstances.

Private Vernon Cook, company "H," 13th Georgia regiment, twenty-one years old, sandy hair, blue eyes, and florid complexion, nervous temperament, enlisted on the 18th May, 1862, in the 11th Georgia regiment. While at second battle of Manassas, he was attacked with rubeola, he took cold by imprudent exposure, and the disease affected his right lung, the pain in which was of constant annoyance, with cough. The lung, however, did not suffer from any serious affection, but his general health became much impaired and debilitated. He was finally discharged from the service. He returned to his home in the State of Georgia, and, after six months, he felt strong enough to re-enlist, which he did in the 13th Georgia regiment.

He enjoyed very good health up to the battle of Chancellorsville. He was severely shocked and knocked prostrate by a shell in that battle. He expectorated much blood, and suffered considerably from pain in his right breast from the effect, and about four days afterwards he became hoarse, and lost the power of speaking above a common whisper—from which he did not recover, but all his other symptoms improved, and he has since then been an inmate of the hospitals He entered this hospital about two months ago, and was one of the patients, unable for active duty, appointed as a guard. He performed his duties for about one week, when he was attacked with pneumonia of the *right lung*, involving quite extensively both lobes. He had considerable fever, and quite a quick and full pulse. Notwithstanding his physical health was feeble, he was treated upon the tartar emetic system, the effect of which occasioned sickness at stomach, with some vomiting. During which feeling of depression he observed, to use his own words, "something broke loose in his breast and run up his throat." He, however, recovered at the time the free use of his speech, with as clear and distinct expression as he ever possessed, and which he still retains, though not entirely recovered from his pneumonia. This patient had been annoyed since the battle of Chancellorsville with *aphonia*, and not until the pneumonia from which he suffered had he been able to obtain any relief, though innumerable remedies had been tried upon him.

Whatever some may think of the seat of aphonia being in the larynx, and involving the vocal chords, as *Wood* explains, yet such a case as this cannot fail to suggest reflections which may aid in throwing additional light on the somewhat obscure pathology of this disease.

